# Fibre-Reinforced Polymers and Steel for the Reinforcement of Wooden Elements—Experimental and Numerical Analysis

**DOI:** 10.3390/polym15092062

**Published:** 2023-04-26

**Authors:** Agnieszka Wdowiak-Postulak, Marek Wieruszewski, František Bahleda, Jozef Prokop, Janusz Brol

**Affiliations:** 1 Department of Strength of Materials and Building Structures, Faculty of Civil Engineering and Architecture, Kielce University of Technology, 25-314 Kielce, Poland; 2 Department Mechanical Wood Technology, Faculty of Forestry and Wood Technology, Poznań University of Life Sciences, Wojska Polskiego 28, 60-637 Poznan, Poland; marek.wieruszewski@up.poznan.pl; 3 Faculty of Civil Engineering, University of Žilina, Univerzitná 8215/1, 010 26 Žilina, Slovakia; frantisek.bahleda@uniza.sk (F.B.); jozef.prokop@uniza.sk (J.P.); 4 Faculty of Civil Engineering, Silesian University of Technology, 44-100 Gliwice, Poland; janusz.brol@polsl.pl

**Keywords:** timber beams, steel plates, FRP composites, strengthening, numerical model

## Abstract

These elements are innovative and of interest to many researchers for the reinforcement of wooden elements. For the reinforced beam elements, the effect of the reinforcement factor, FRP and steel elastic modulus or FRP and steel arrangement of the reinforcement on the performance of the flexural elements was determined, followed by reading the load-displacement diagram of the reinforced beam elements. The finite element model was then developed and verified with the experimental results, which was mainly related to the fact that the general theory took into account the typical tensile failure mode, which can be used to predict the flexural strength of reinforced timber beams. From the tests, it was determined that reinforced timber beam elements had relatively ductile flexural strengths up to brittle tension for unreinforced elements. As for the reinforcements of FRP, the highest increase in load-bearing capacity was for carbon mats at 52.47%, with a reinforcement grade of 0.43%, while the lowest was for glass mats at 16.62% with a reinforcement grade of 0.22%. Basalt bars achieved the highest stiffness, followed by glass mats. Taking into account all the reinforcements used, the highest stiffness was demonstrated by the tests of the effectiveness of the reinforcement using 3 mm thick steel plates. For this configuration with a reinforcement percentage of 10%, this increase in load capacity was 79.48% and stiffness was 31.08%. The difference between the experimental and numerical results was within 3.62–27.36%, respectively.

## 1. Introduction

Wood is a natural composite material, the same as some modern fibre-reinforced polymers (FRPs). Wood fibres consist of cellulose embedded in a matrix of hemicellulose and lignin [1,2]. The tensile strength along the wood fibres is one of the important mechanical properties [1,3]. The mechanical properties of wood make it suitable for use as structural elements in the roof as well as floor systems. It is worth noting that traditional building structures in Europe and the world were usually constructed with floor systems made of unidirectional timber floors supported on softwood or hardwood joists and roof frames made of timber trusses [1]. Timber beams were typically used on the ground floor, also to fill the space above the room and provide structural support. These timber structures were usually subjected to bending (lateral) loads. It is important to note that timber structural members are used in centuries-old, historic, or heritage buildings, as well as in newer structures built around the world.

Wood and its mechanical properties in structural elements are determined by a number of structural and geometric features, including the presence of natural defects such as knots or cracks. In addition, wooden structures are characterised by the fact that they can be weakened under adverse conditions and show signs of ageing during long-term use. Consequently, in the case of historic wooden buildings, their structural elements usually need to be repaired and reinforced. As well as in modern timber buildings, with today’s ever-increasing demands on the strength, or stiffness, of their structural elements would also facilitate the use of reinforcement technology [4]. It should also be noted that timber elements reinforced with steel materials have been extensively studied in recent decades (e.g., refs. [5,6]). However, their use has been limited due to their relatively high density and poor corrosion resistance in relation to other reinforcing materials. Recently, therefore, there has been an increasing amount of research into the reinforcement of timber structures with fibre-reinforced polymer (FRP). This is due to the fact that these fibre-reinforced polymers have excellent corrosion resistance, a high strength-to-weight ratio and a variety of FRP products [7]. 

In addition, the use of fibre-reinforced polymer (FRP) composite materials may provide solutions to improve the inferior mechanical properties of timber elements [8,9,10,11]. Furthermore, fibre-reinforced polymers (FRPs) have been used extensively over the past two decades to renovate and reinforce existing structures. In the case of glass or carbon FRPs, they exhibit a high strength-to-weight ratio, corrosion resistance and provide design flexibility [8,12,13,14]. The usual FRP composites used as reinforcement for timber beams are carbon FRP (CFRP), E-glass FRP (GFRP) and aramid FRP (AFRP) [15,16,17,18,19,20]. It should be noted that the production processes for these fibres are, however, energy-intensive, while the initial costs are still high. In recent years, natural mineral-based FRP components such as basalt FRP (BFRP), among others, have been proposed. Well, BFRP is characterised by low cost; high fire resistance; and good thermal, electrical and acoustic insulation properties [21,22,23,24]. As well as basalt fibre has high tensile properties (e.g., tensile strength 1850–4800 MPa). In contrast, glass fibre, like basalt fibre, requires a large amount of energy to produce due to the high melting point of basalt rock (1300–1700 °C). In addition, eco-friendly and economical FRPs of plant origin (e.g., flax, jute, yucca) have been introduced as an alternative to glass, carbon and basalt fibre materials [8,25]. Studies of plant fibres (e.g., flax) have shown that, as a single fibre, they have comparable specific mechanical properties (e.g., specific tensile strength and stiffness) compared to man-made glass fibres [8]. However, this is misleading, as the length of natural fibres is limited when a carbon or glass fibre can be manufactured to have infinite length [26,27,28]. Fibre-reinforced wood composites have found wide application in many industrial fields. They are particularly popular because of their lightweight, high strength, excellent corrosion resistance and fatigue resistance, studies show the high performance and advantages of FRP [29,30].

For board-shaped FRP materials (e.g., slabs or sheets), the external bonding method (EBM) is widely used due to its convenience. As well as glued laminated timber beams, LVL beams or CLT beams also allow for the horizontal or vertical embedding of slab-shaped FRP in wood laminates during the gluing process [4]. In [8], the authors tested eight full-size laminated timber beams, consisting of both unreinforced beams and beams reinforced with externally glued CFRP sheets. From the tests, it was found that the flexural strength of the laminated glulam beams reinforced improved significantly as the width, length or thickness of the CFRP bonding increased. On the other hand, it should be remembered that in the case of FRP bars or FRP ropes or prestressing tendons, manufacturing procedures always require grooving on the wood surface. In work [31], pultruded rectangular CFRP bars were investigated as reinforcement for glued laminated beams and the effect of the anchorage length of the CFRP bars on the bending of the beams was determined. Experimental studies in papers [32,33] based on flexural strength tests of timber beams with different dimensions, with different glued-in FRP fibres (i.e., CFRP cords, GFRP cords and BFRP bars) and with two types of adhesives (i.e., melamine glue and epoxy resin). The study concluded that FRP cord reinforcement is a potential alternative to pultruded bars. In another paper [34], the research was based on the investigation of pure laminated bamboo beams, and then the laminated bamboo beam was reinforced with both unstressed and pre-stressed BFRP rods. From the tests, it was determined that the prestressed beams did not show an improvement in ultimate load capacity compared to the unstressed beams. 

It should be noted that changes over time in the strength or stiffness properties of composite members vary according to the environment in which they operate. It must be remembered that different environmental conditions affect the bars before they are incorporated into the structure and others during or after incorporation. Well, the aggressiveness of the environment varies depending on the place of incorporation of an element reinforced with FRP bars. Therefore, the properties of the bars may deteriorate, improve or remain the same. Environmental factors that affect the durability of FRP bars include water, ultraviolet radiation, elevated temperature, acid solutions, alkaline solutions, salt solutions, heating and cooling cycles, freezing and thawing cycles, stresses in the bar. The durability of composite bars is greater than that of steel due to the polymer resin that forms the surface layer of the bar, as it is the one in direct contact with the external environment. 

The following work specifies a study of the reinforcement of wooden components with mats and strips carbon CFRP, aramid AFRP, glass GFRP strips, steel/BFRP bars and steel plates. The main purpose of this work was to determine the effectiveness of the applied reinforcement and to check the difference in the effectiveness of this reinforcement between FRP and steel. Therefore, research elements with similar mechanical properties were introduced to this work along with an analysis of the structural and geometrical features of the beams in order to provide the most satisfactory reinforcement configuration, together with the presentation of reliable reinforcement efficiencies along with the analysis of the pattern, arrangement and percentage of the reinforcement used. An important issue of the presented research is the assessment of the influence of various reinforcing materials on the strength of wooden beams. The aim of the article is to assess the contribution of reinforcement to changes in the modelling of structural beams using numerical methods. Numerical techniques, being much cheaper because they require less experimental research, are also environmentally friendly. The tests were aimed at indicating the accuracy of modelling in the case of large-size beams in order to assess some of the properties of the applied reinforcements with higher fire resistance or higher corrosion resistance in the future.

## 2. Materials and Methods

### 2.1. Timber

The solid timber elements were made of Scots pine (*Pinus sylvestris* L.). All mechanical properties were tested per EN 408 [35] and are summarised in Table 1. All structural and geometrical characteristics were determined per PN-D-94021 [36] and classification per EN 338 [37]. During testing, an average density of 711.05 kg/m^3^ was obtained with a coefficient of variation (V) of 11.95%; meanwhile, the average moisture content was 10.46% with a V of 4.68%.

### 2.2. FRP and Steel

The paper shows experimental studies, which were compared with numerical results. These studies consisted of 50 unreinforced solid beam elements and 195 externally reinforced solid beam elements reinforced with steel plates, steel/FRP bars, carbon fibre-reinforced polymer (CFRP), aramid fibre-reinforced polymer (AFRP), glass fibre-reinforced polymer (GFRP) mats or fabrics, were tested under four-point bending loading.

For the reinforcement of the timber elements, the following were used:

- C1 timber elements—SikaWrap^®^-230 C carbon mats [38], (Sika Poland Sp. z o.o., Warsaw, Poland): laminate tensile strength 3500 MPa, tensile modulus 225 GPa; pieces 15; reinforcement grade 0.43%.
- C2 and C3 wood elements—carbon tapes, which were produced based on carbon fibres from the manufacturer TORAYCA (Surfpol, Nowy Kurzeszyn, Poland) type T700S (density 1.8 g/cm^3^, tensile strength 4900 MPa, tensile modulus 230 GPa); units of 30; reinforcement grade 1.67%.
- A1 wood elements—S&P aramid mats [39], (S&P Polska Sp. z o.o., Malbork, Poland), A-Sheet 120 290 g/m^2^, modulus of elasticity ≥ 120 kN/mm^2^, tensile strength ≥ 2900 N/mm^2^; pieces 15; reinforcement grade 0.67%.
- A2 and A3 wood elements—aramid tapes, which were produced based on aramid fibres from the manufacturer Kevlar 49 DuPont TM (Surfpol, Nowy Kurzeszyn, Poland), (density 1.44 g/cm^3^, tensile strength 3600 MPa, tensile modulus 124 GPa); pieces 30; reinforcement grade 1%.
- S1, S2 and S3 wood elements—S&P glass mats [40], (S&P Polska Sp. z o.o., Malbork, Poland). G-Sheet E 50/50 350 g/m^2^ with a modulus of elasticity ≥ 73 kN/mm^2^ and a tensile strength ≥ 3400 N/mm^2^, pieces 45; reinforcement grade 0.22%.
- ST1—steel plates (Carbon steel, thickness 3 mm, resistant to high temperatures, tensile strength in the range of 485–620 MPa and the minimum yield strength should exceed 260 MPa) pieces 15; reinforcement grade 10%.
- ST2—steel plates (S355J2, thickness 2 mm, structural sheets in accordance with EN 10025). The steel is well weldable. It is suitable for machining and has higher corrosion resistance—pieces 15; reinforcement grade 6.67%.
- P1—steel bars (diameter 2 mm steel plain bar, S235JR), pieces 15; reinforcement grade 1.14%.
- P2—BFRP bars (diameter 2 mm, elasticity modulus 52.8 GPa, tensile strength 1185 MPa), pieces 15; reinforcement grade 1.14%.

### 2.3. Adhesive

The epoxy resin-based adhesive layer—GRM Systems s.r.o. (Olomouc, Czech Republic)—was obtained by mixing epoxy resin LG 385 (density 1.18 ÷ 1.23 g/cm^3^, viscosity 600 ÷ 900 mPa·s) with hardener HG 385 (density 0.94 g/cm^3^, viscosity 50 ÷ 100 mPa·s). After mixing the resin and hardener, the adhesive achieved a bending strength of 110 ÷ 120 MPa and a modulus of elasticity of 2700 ÷ 3300 MPa. To achieve maximum strength according to the manufacturer GRM Systems s.r.o. (Olomouc, Czech Republic), annealing was necessary.

### 2.4. Specimen Preparation

**Figure 1:**
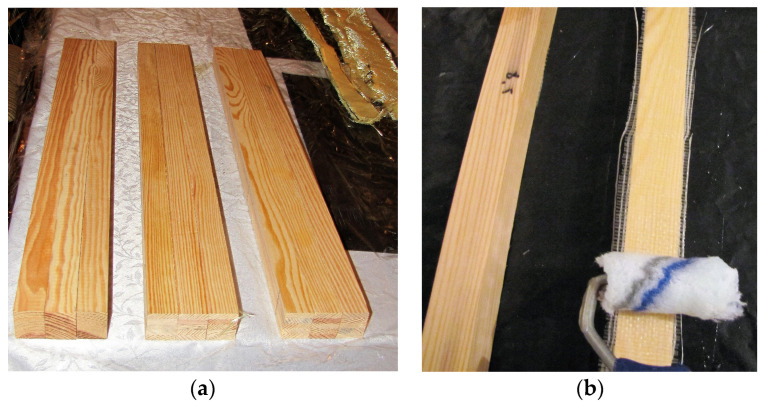
A diagram of the preparation of timber components using FRP: (**a**) cleaned wooden parts. (**b**) application of epoxy adhesive and glass matting.

Figure 1 illustrates a scheme for the preparation of the reinforcement of the timber elements with plates, mats and carbon, aramid and glass tapes (dimension 30 × 30 × 600 mm). The manufacturer’s technical specifications for the fibre composites used are presented in Table 1b.

For the reinforced beams, all fabric and 600 mm long FRP mats and steel plates were embedded horizontally on the tension side of the beams. The FRP-reinforced timber elements were manufactured using the following procedures:

- Preparation of the timber elements by drying and cleaning the surface;
- Gluing the FRP and steel reinforcement on the designated element;
- Gluing, pressing under pressure and conditioning of the beam samples;
- Sanding the surface of the beams to remove residual glue once the curing strength has been reached.

In addition, basalt and steel rods with a diameter of 2 mm were placed in the square grooves and sealed with epoxy glue.

One group of unreinforced timber beams was used as reference specimens in this study, as shown in Table 1. The other thirteen groups of timber elements were designed to investigate the effect of the reinforcement factor steel, CFRP, BFRP AFRP and GFRP and a given reinforcement system on the bending of the reinforced timber beams. 

**Figure 2:**
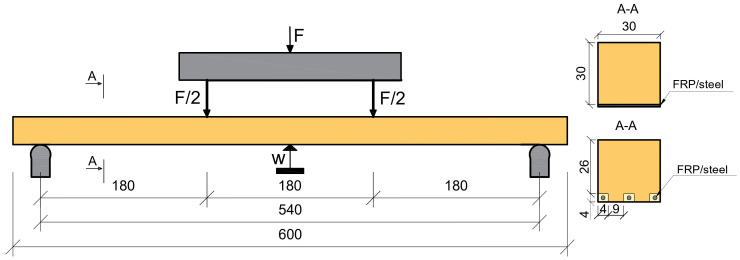
Set-up of the four-point test [35].

The degree of FRP and steel reinforcement is calculated as the ratio of the cross-sectional area of the FRP and steel reinforcement to the cross-sectional area of the beam. A detailed analysis of the degree of reinforcement is described in Section 2.2—FRP and Steel. Figure 2 shows the reinforcement scheme for timber members.

### 2.5. Methods

The bending strength test was carried out based on PN-EN 408 [35]. The static bending strength of the timber elements was determined using a ±250 kN electromechanical testing machine (Zwick). The test specimens were loaded symmetrically with two concentrated forces at a span equal to 18 times the beam’s cross-sectional height. The load was applied at a constant feed rate so that the maximum load was achieved after (300 ± 120 s). The loading speed of the specimen was determined by preliminary tests. The optimum time to reach F_max_ was 300 s. The loading speed during static bending tests was 0.075 mm/s.

The bending strength was determined based on PN-EN 408 [35].

Thirteen groups of 15 reinforced solid wood beams and 50 pcs unreinforced beams were tested using a four-point bending test configuration, as shown in Figure 2. 

The details of the specimens are shown in Table 1. The test pieces had a cross-sectional dimension of 30 mm × 30 mm. The length of the beam elements was 600 mm.

The material parameters of wood, FRP materials, steel bars and steel plates in numerical tests were adopted on the basis of own research (Table 1b) and literature data [38]. In experimental studies, only the modulus of elasticity along the E_x_ fibres was determined. Poisson’s coefficients υ (Table 1b) for wood were adopted in accordance with [41] other values were determined on the basis of PN-EN 338 [37]. Technical values for the applied reinforcements and epoxy adhesive were determined on the basis of the manufacturer’s data. The remaining data were automatically recalculated in the ANSYS environment. The wood and reinforcements were defined as orthotropic materials and the glue as an isotropic material.

**Figure 3:**
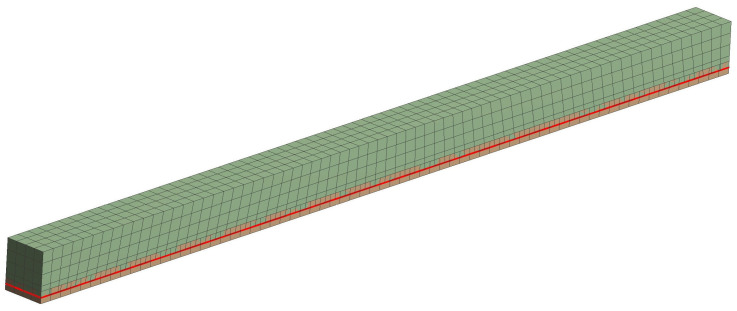
FEM model introduced to the program—ST1.

The numerical analysis for unreinforced and reinforced timber elements was carried out on the basis of the Finite Element Method in the ANSYS 16.0 Static Structural module. All FEM models for all groups of unreinforced and reinforced timber elements were developed in the program to simulate the static bending work analysis. The model was created with the entire solid part as a timber element. However, for the reinforced models, an additional shell part was introduced as reinforcement in the form of fabric or FRP mats, strips, bars and steel plates. For modelling, the geometrical dimensions of the SEM models were the same as those of the wooden elements in the experimental analysis. The finite element mesh consisted of hexa- and tetragonal elements. Wood was modelled as hexagonal elements with a dimension of 10 mm. Due to the small dimensions of the reinforcement and epoxy glue in relation to the remaining geometry. They were defined as tetragonal elements with a size equal to 5 mm. An example model is shown in Figure 3.

## 3. Results and Discussion

The results of the bending strength tests for solid timber elements reinforced with steel plates, basalt bars, carbon, aramid and glass mats are shown in Table 2. From the tests, it is noted that the highest mechanical properties for FRP were obtained for the basalt fibre-reinforced timber specimens, while the lowest was obtained for the aramid fibre-reinforced timber elements. It should also be noted that for these degrees of reinforcement, the results for the reinforced elements are comparable. However, considering all the steel and FRP-reinforced timber elements, the best mechanical properties were obtained for the steel plate reinforcement. A thicker steel plate allowed for obtaining better indicators of increase in load capacity and stiffness.

**Figure 4:**
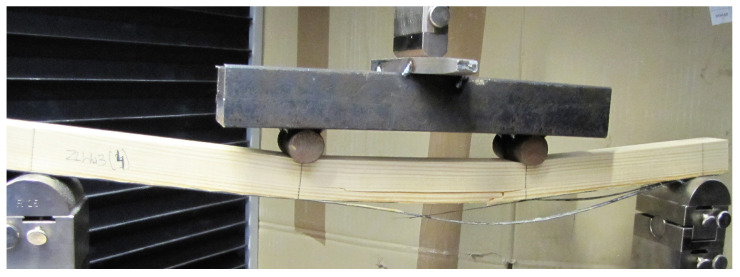
Image of the destruction of the sample C1.

Based on the tests, it was found that for all the timber elements, failure occurred due to cracking in the tensile zone (Figure 4). Usually, failure occurred due to brittle cracking of the knots located in the tension zone of the samples. On the other hand, in most cases for the quality grade of the wood, failure occurred with a longitudinal continuous crack formed along the grain line of the wood, after which the crack is propagated. Only in some specimens did the carbon matting, the webbing or aramid matting and the glass matting break. From the tests, it was noted that a failure pattern from brittle failure in the tension zone to more ductile failure is possible for any type of reinforcement when increasing the degree of FRP reinforcement. Furthermore, the destruction of the steel-reinforced timber elements did not result in the destruction of the steel elements. The results obtained confirm the observations of other researchers, indicating the accumulation of stresses in the zone of elements with a heterogeneous structure as wood [41,42].

### 3.1. Load–Deflection

**Figure 5:**
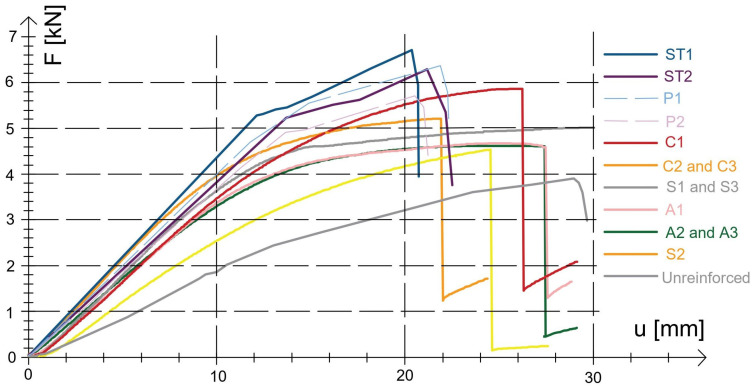
Load-deflection curve for all reinforced timber elements.

The ‘load-deflection’ curves for all timber elements are shown in Figure 5.

It can be seen that in some of the wooden elements the load dropped suddenly after the final load strength was reached, which may show that brittle fracture occurred in these wooden elements in bending. For stiffness, the highest stiffness if we consider the type of material FRP was achieved by carbon mats, followed by glass mats. In contrast, aramid fibres had a lower stiffness. It should be noted that the use of steel plates satisfactorily improved the load-bearing capacity and stiffness of the wooden elements, even in the case of ST1 reinforcement, load-bearing capacity increased by 79.48% and stiffness by 31.08%. However, in the case of reinforcement with ST2 steel plates, this load capacity increased by 57.14% and stiffness by 29.73%. However, for steel bars, this load capacity increased by 59.74% and stiffness by 31.08%. The degree of reinforcement was defined as the percentage increase in strength of reinforced beams relative to unmodified reference beams (Table 2) [28,41]. Determined as the cross-sectional area without reinforcement in relation to the cross-sectional area of the reinforcement.

### 3.2. Comparison of Experimental and Numerical Models Depending on the Type of Reinforcement and Degree of Reinforcement

**Figure 6:**
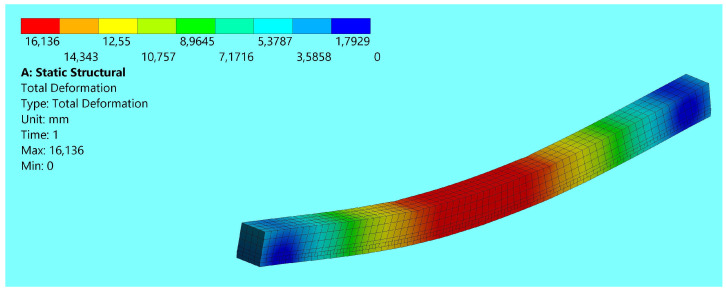
Displacement results for the ST-1 wooden beam at maximum load.

The numerical analysis carried out for both unreinforced and reinforced wooden elements was based on the finite element method for static bending work. An important element of model differentiation was the separation of parts of wooden elements, and an additional shell part was introduced for reinforced models. Modelling results for similar geometric dimensions of SEM models and wooden elements in the experimental analysis showed similar strength relationships. Despite the small dimensions of the reinforcement and epoxy glue in relation to the rest of the geometry of the beams, significant differences in the achieved strength ratios were obtained, as confirmed by the study [16,17,21,42,43,44]. Figure 6 shows the results of displacements for the ST-1 wooden beam. A comparison of the experimental and numerical results is shown in Table 3.

Based on experimental tests, the highest increase in load-bearing capacity was for steel plates 79.48%, while the lowest was for glass mats at 16.62%. As far as tapes are concerned, the highest for carbon straps was 35.06% and the lowest for aramid tapes was 18.96%. As for the mats, the highest values were obtained by the carbon mat and the lowest by the glass mat. Steel bars, on the other hand, had slightly higher load-bearing capacity values than basalt bars, and basalt bars again turned out to be slightly more rigid. The best reinforcement efficiency was demonstrated by the use of steel sheets. It should be noted that for structural design, the degree of reinforcement is important. The highest degree of reinforcement for FRP here is for carbon tapes—this increase is 35.06%—and the lowest is for glass mats—this increase is significant at 16.62% and 35.58%. The flexural strength of the wood beams can be significantly improved by reinforcing the mats or tapes with CFRP, GFRP, AFRP and bars BFRP due to the fact that no premature failure of the FRP occurred during the experimental tests, as well as no failure between the adhesive and the FRP layer. Too strong a degree of reinforcement had a slight effect on the increase in strength. The accuracy of the insertion of the reinforcement into the timber beams is also of great importance [45,46]. In the case of steel elements, the highest reinforcement process was shown by 3 mm thick sheets. This reinforcement showed the best reinforcement efficiency parameters.

Publications have presented studies on the use of reinforced beams (GFRPP, SCFM CFRP, AFRP and FRP) as reinforcement in concrete structures and have also demonstrated improved strength properties. In this paper, a comparative evaluation of wooden beams significantly different from concrete was carried out, examining the experimental and numerical strength. However, it can be expected that, as for traditional reinforced beams, the hydrothermal environment accelerates the development of damage, which is mainly related to the degradation of fibres and resin. At the same time, numerous studies indicate the influence of modern stem resins that increase the bond between wood materials and applied reinforcements [47,48].

Significant variability in the obtained modelling results is a direct result of the structure of structural wood. Unlike homogeneous materials (concretes), wood exhibits significant anisotropy of structure and variability in the severity of defects. Numerous studies show a strong correlation between the severity of basic defects in wood on the variability of strength. This is reflected in the results of the study [49,50].

Compared to the differences in the experimental and numerical models for unmodified beams, the differences reached 30%. When comparing the experimental and numerical models of modified beams, there is a good correlation between deflections. For differences in deflection correlation for experimental and numerical models of modified beams, the differences were from 3% to 23%. The difference is the lowest for tapes and glass-mat-reinforced elements. In general, the numerical models are perfectly applicable to the design of timber structures.

Previous research on modifying wooden beams to improve their stiffness and strength is subject to model fitting errors. Numerous works point to the need to clarify the factors affecting the limits of modified beam models with more complex structural solutions. The research challenge is to take into account fasteners and interference in the construction of modified beams during the assembly stage of building structures [29,51].

## 4. Conclusions

The study examined the flexural strength of solid wood beams unreinforced and reinforced with CFRP, AFRP and GFRP strips, mats and steel, BFRP bars and steel plates. In addition, the evaluation of the effectiveness of reinforcements with different proportions of reinforcement on the static work of wooden beams was studied. The static work of wooden members analysed experimentally was related to numerical analysis.

- Increased reinforcement effectiveness was obtained for elements reinforced with FRP and steel materials with higher MOE.
- Wooden beams fail due to cracks occurring in the tension zone. In unreinforced elements, these were mainly cracks occurring in the deformation zone (the most numerous defects were knots). In reinforced elements, it was damage occurring in the compression zone due to gradual crack propagation and crushing.
- The largest increase in load-carrying capacity was confirmed for steel plate reinforcement by 79%, while the smallest for glass mats and was only 16%. It should be noted that tests conducted for full-size elements increase the severity of defects since wood is a heterogeneous material. In full-size wooden beams, despite the selection of wood of the same quality and strength class, tests can have significant scatter due to the anisotropy and variability of wood defects.
- Taking into account the cost of FRP material, environmental friendliness and high resistance to corrosion and high temperatures, very high parameters were obtained for BFRP bars—an increase in load capacity by as much as 50% and stiffness by 30%.
- High and satisfactory results were confirmed for high-temperature and corrosion-resistant steel reinforcement elements—load-carrying capacity increases of 79–57% and stiffness increases of 31–29%.
- Numerical models allow for obtaining approximate results as experimental tests. The difference of 3% to 22% is due to the heterogeneity of the wood material, such as permissible knots, cracks o deviations of wood fibres.

## Figures and Tables

**Table 1:** (**a**) Mechanical properties of unreinforced timber beams. (**b**) Mechanical properties of wood EN 338 [37] and reinforcing materials introduced into the numerical model.

(**a**)									
**Mechanical Property Parallel to Grain**	**Pieces**	**Size (mm)**		**Average Strength (MPa)**			**SD** **(MPa)**	**V_s_ (%)**	
Tensile strength	50	30 × 30 × 600		77.48			16.01	10.66	
Compressive strength	50	30 × 30 × 180		48.70			7.23	8.84	
(**b**)									
**Materials**	**MOE (MPa)**			**Poisson’s Ratio**			**G (MPa)**		
	**X**	**Y**	**Z**	**X**	**Y**	**Z**	**XY**	**YZ**	**XZ**
Wood KW (C35)	13,000	430	430	0.54	0.027	0.54	810	81	810
Epoxy glue	3300	109.16	109.16	0.3	0.015	0.3	-	-	-
C1	225,000	7442.94	7442.94	0.28	0.014	0.28	-	-	-
C2 and C3	230,000	7608.34	7608.34	0.28	0.014	0.28	-	-	-
A1	120,000	3969.57	3969.57	0.36	0.0180	0.36	-	-	-
A2 and A3	124,000	4101.89	4101.89	0.36	0.0180	0.36	-	-	-
S1, S2 and S3	73,000	2414.82	2414.82	0.22	0.0110	0.22	-	-	-
ST1	210,000	6946.74	6946.74	0.25	0.0125	0.25	80,000	8000	80,000
ST2	210,000	6946.74	6946.74	0.25	0.0125	0.25	80,000	8000	80,000
P1	210,000	6946.74	6946.74	0.25	0.0125	0.25	80,000	8000	80,000
P2	52,800	1746.61	1746.61	0.19	0.0095	0.19	-	-	-

**Table 2:** Flexural strength test results—experimental and numerical analysis [36,38,39,40].

The Symbol/Description of the Sample	The Beam Density (kg/m^3^)	Moisture (%)	Wood Annual Ring (mm)	F (kN)	f_m_ (MPa)
**P1**—steel bars—S235JR. wood quality class C35 (KW), fibre twist (2–3%), Heartwood, proportion of earlywood and latewood	679.21	7.90	1.04	6.15	123.00
**P2**—BFRP bars, wood quality class C35 (KW), proportion of earlywood and latewood and latewood	681.32	8.15	1.98	5.78	121.00
Averages Series P	680.27	8.03	1.51	5.97	122.00
S	1.49	0.18	0.66	0.26	1.41
SD	1.28	0.13	0.47	0.19	1.00
Vs	4.09	1.41	2.06	2.15	3.12
**ST1**—steel plates—carbon steel. wood quality class C35 (KW), fibre twist (2–3%), heartwood, proportion of earlywood and latewood	686.81	8.30	1.92	6.91	138.20
**ST2**—steel plates S355J2. wood quality class C35 (KW), proportion of earlywood and latewood and latewood	678.25	8.60	2.01	6.05	121.00
Averages Series ST	682.53	8.45	1.97	6.48	129.60
S	17.56	0.21	0.06	0.61	12.16
SD	4.28	0.15	0.05	0.43	8.60
V_s_	1.19	4.25	7.17	6.55	6.22
**C1**—carbon mat SikaWrap^®^-230 C wood quality class C35 (KW), fibre twist (2–3%), heartwood, proportion of earlywood and latewood	696.99	8.10	1.80	5.87	117.41
**C2**—carbon tapes wood quality class C35 (KW), proportion of earlywood and latewood and latewood	679.22	8.70	2.07	5.19	103.72
**C3**—carbon tapes wood quality class C35 (KW), Heartwood, significant sapwood, proportion of earlywood and latewood	661.86	9.10	1.9	5.21	104.30
Averages Series C	679.35	8.63	1.90	5.40	108.47
S	17.56	0.50	0.16	0.39	7.74
SD	11.75	0.36	0.11	0.30	5.96
V_s_	2.59	5.83	8.47	7.14	7.14
**A1**—aramid mat A-Sheet 120 290 g/m^2^ wood quality class C35 (KW), fibre twist (1–2%), heartwood, significant sapwood, proportion of earlywood and late wood	650.00	8.60	1.70	4.67	93.45
**A2**—aramid tapes. wood quality class C35 (KW), fibre twist (0.5–1%), heartwood, sapwood, proportion of early and late wood	655.56	8.90	1.20	4.62	92.39
**A3**—aramid tapes, wood quality class C35 (KW), wavy grain, Heartwood, Sapwood, proportion of earlywood and latewood	712.96	8.80	3.10	4.54	90.74
Averages Series A	672.84	8.77	1.98	4.61	92.19
S	34.86	0.15	1.00	0.07	1.36
SD	26.75	0.11	0.75	0.05	0.97
V_s_	5.18	1.74	50.78	1.48	1.48
**S1**—glass mat type G-Sheet E 50/50 350 g/m^2^ wood quality class C35 (KW), wavy grain, heartwood, significant sapwood, proportion of earlywood and latewood and late	709.26	7.70	1.60	5.34	106.82
**S2**—glass mat type G-Sheet E 50/50 350 g/m^2^ wood quality class C35 (KW), heartwood, sapwood, proportion of earlywood and latewood	655.56	8.50	1.70	4.49	89.77
**S3**—glass mat type G-Sheet E 50/50 350 g/m^2^ wood quality class C35 (KW), bark, heartwood, sapwood, proportion of earlywood and latewood	694.44	7.90	1.60	5.10	102.07
Averages Series S	686.42	8.02	1.67	4.98	99.55
S	27.74	0.40	0.05	0.44	8.79
SD	20.58	0.30	0.04	0.33	6.52
V_s_	4.04	4.96	3.11	8.83	8.83

f_m_—flexural strength (N/mm^2^). F—load (N).

**Table 3:** Comparison of experimental and numerical results.

Symbol	Type of Reinforcement	The Degree of Reinforcement (%)	Experimental Model—Destructive Force (kN)	Experimental Model—Maximum Deflection (mm)	Numerical Model—Maximum Deflection (mm)	Difference (%)
Unreinforced	-	0.00	3.85	29.6	21.50	27.36
P1	bars	1.14	6.15	21.8	17.56	19.45
P2	bars	1.14	5.78	20.6	17.02	17.38
ST1	plate	10.0	6.91	20.4	16.14	20.88
ST2	plate	6.67	6.05	20.8	16.58	20.29
C1	mats	0.43	5.87	26.2	20.20	22.90
C2 and C3	tapes	3.33	5.20	21.9	18.42	15.89
A1	mats	0.67	4.67	25.2	20.38	19.13
A2 and A3	tapes	4.00	4.58	24.9	20.19	18.92
S1 and S3	mats	0.22	5.22	30.0	23.71	20.97
S2	mats	0.22	4.49	24.6	23.71	3.62

## Data Availability

All data generated or used during the study appears in the submitted article.
